# Diffusive model to assess the release of chemicals from a material under intermittent release conditions

**DOI:** 10.1038/s41598-022-07144-0

**Published:** 2022-03-02

**Authors:** Diego Frezzato, Gianluca Stocco, Enrico Boscaro, Marco Ferraro, Andrea Tapparo

**Affiliations:** 1 Department of Chemical Sciences, University of Padua, Via Marzolo 1, 35131 Padua, Italy; 2 Normachem S.r.l., Via Roma 14, 35014 Fontaniva, Padua, Italy; 3 Department of Pharmacy, University of Naples Federico II, Via D. Montesano 49, 80131 Naples, Italy

**Keywords:** Chemical safety, Method development

## Abstract

We consider the archetype situation of a chemical species that diffuses in a material and irreversibly escapes through the interface. In our setup, the interface switches between two states corresponding to ‘release phase’ (absorbing boundary) during which the species is released to the exterior, and ‘pause phase’ (reflecting boundary) during which the species is not released and its concentration profile inside the material partially relaxes back to uniformity. By combining numerical solution of the diffusion equation and statistical analysis of the outcomes, we derive upper and lower bounds and an empirical approximation for the amount of species released up to a certain time, in which the only information about the release-pause alternation schedule is the number of release phases and the average duration of a release phase. The methodology is developed thinking especially to dermal exposure assessment in the case of a slab-like homogeneous material irreversibly releasing chemicals during a number of contacts. However, upon proper extensions, this approach might be useful for inspecting other situations that are encountered, for instance, when dealing with leakage of chemicals in environmental contexts and regulatory toxicology.

## Introduction

Characterizing the release of chemicals from a material through the interface is a crucial problem in disparate situations. Just to mention a few, think to environmental ambits in which the species is released from the material into a different phase (e.g., volatile species released in the air from floors^1^ or other building materials^2,3^, or released from buildings into receiving water under wet weather conditions^4^), in the risk assessment of near-field exposure to consumer products^5^ (e.g., release of chemicals from consumer articles^6^ and cotton wipes^7^ into derm, from plastics into drinking water^8^, from packaging films into food^9^), and in the context of controlled drug release from pharmaceutical devices^10,11,12^.

Although the leakage is typically continuous, in some cases it might be intermittent with alternation of release-pause phases. For instance, chemicals in consumer objects are intermittently released to the derm through repeated contacts of limited duration; just think to rings or earrings releasing Nickel while they are worn, to a baby that puts a toy in the mouth from time to time, to phthalates released from plastic handles when the tools are used, etc. Yet, containers that release chemicals only when they are filled with liquids, or species that might pass into the environment from building materials or abandoned waste only under wet weather conditions. In all these examples, the release is intermittent and the (partial) redistribution of the species inside the material between two consecutive phases might affect, to some extent, the subsequent release rate. An accurate assessment of the amount of the released chemicals should make use of appropriate models, and the outcomes could be relevant for the risk assessment in occupational medicine, in REACH Regulation, or in studies of exposure to environmental pollutants. Despite the potential relevance of the issue, to our knowledge a systematic quantitative analysis is still lacking. Such analysis would be clearly case-dependent, hence to start facing this topic we must select a specific relevant situation.

Among the variety of contexts in which the intermittent release from a material into a hosting phase is important, here we focus on simple but relevant situations like that of a consumer material, or a tool, which comes into contact with the skin. We shall move the first steps in the release assessment by treating the simplest but realistic scenario that allows us to make basic modeling and numerical explorations. Specifically, we will assume that the release is irreversible and controlled by the diffusion in the material. A crucial target is to evaluate the total quantity $m_{\mathrm{ext}}$ of species released through the interface up a certain time $t_{\mathrm{tot}}$, given that the system is initially at equilibrium. How is $m_{\mathrm{ext}}$ related with the diffusivity of the species in the bulk material, with the geometrical features of material and interface, and especially with the history of the contacts between material and exterior?

To tackle the problem on theoretical grounds, it proves convenient to consider the simplest archetype setup of a slab-like homogeneous material with given diffusion coefficient of the chemical species in it, and with uniform volumetric concentration at the initial equilibrium. The slab geometry is the simplest and natural setup if we refer to macroscopic objects whose exposed surface, from the viewpoint of the diffusion processes, looks locally planar. Assuming a constant diffusion coefficient is the most natural choice if the material is meant to remain uncontaminated by the external medium during the contact phases and if its homogeneity is preserved (a typical example is that of leakage of additives from plastics in the rubbery state, to which these assumptions are normally applied). The homogeneous initial load is also the most common situation thinking to such a kind of material after a preceding long resting phase. In addition, it is assumed that the chemical species passes irreversibly through the interface treated as a perfect absorbing boundary. This means to refer to the situation in which the external medium is quickly renewed at the interface so that the external concentration of the species can be assumed to be vanishingly small. Based on these assumptions, as stated above our purpose is to work out bounds on $m_{\mathrm{ext}}$ and even a likely approximation getting rid of the details of the release-pause schedule. Facing this problem goes much beyond the mere solution of the diffusive model at given schedule. The critical (and novel) aspect, in fact, consists in devising a global statistical synthesis of the schedule-dependent outcomes. This is precisely the target of the present theoretical-methodological work.

We proceed as follows. In “Theoretical model and approach” Section we present the theoretical model and our approach. “Results” Section presents the results; the methodological details and the analysis that support the findings are provided in “Methodological aspects and analysis” Section. First we treat the case of a releasing material of infinite thickness (“Infinite thickness” Section), for which we derive lower and upper bounds on $m_{\mathrm{ext}}$ and, more importantly, an empirical approximation. The extension to the finite thickness case is done later (“Finite-thickness correction” Section) through the introduction of a correction factor on the upper bound. Section “Remarks and perspectives” is devoted to final remarks and perspectives. We must stress that the methodology that we are going to illustrate is applicable to any situation that adheres to the physical assumptions of the model.

## Theoretical model and approach

Let us consider a planar slab-like material in which the chemical species has a constant diffusion coefficient *D*. Let *A* be the contact area with the exterior, and *b* the thickness of the material. Let us also assume that the chemical species is initially uniformly distributed in the material with volumetric concentration $c_0$.

**Figure 1:**
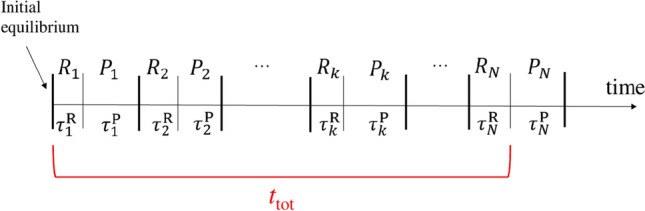
Scheme of the release-pause phases.

We focus here on the case in which the leakage is intermittent, that is, there is a number *N* of release phases (R) separated by pause phases (P) during which the material does not release the species and the interface behaves as a reflecting boundary. Figure 1 depicts the operation schedule on the time axis.

Concerning $m_{\mathrm{ext}}$ at the time $t_{\mathrm{tot}}$, our aim is to provide useful lower and upper bounds and an approximation in terms only of the parameters *D*, *A*, *b*, $c_0$, and of the number *N* of release phases. In this perspective, the specific features of the operation schedule should not enter explicitly. To achieve the target, we need first to simulate a large ensemble of instances (each corresponding to a specific schedule) and get the exact values of $m_{\mathrm{ext}}$; the subsequent step is to make a global analysis of the outcomes to see if useful indications about $m_{\mathrm{ext}}$ do emerge at fixed values of the few parameters listed above.

Given a specific release-pause schedule, the exact treatment of the problem requires to solve the diffusion equation for determining the evolution of the concentration profile inside the material. In fact, as shown below, the release rate from a unit-area portion of interface is proportional to the instantaneous slope of the concentration profile at the interface.

Let us take as *x*-axis the direction orthogonal to the interface and pointing towards the interior of the material. At distance *x* from the interface, *c*(*x*, *t*) is the concentration of the species at the time *t*. Let *J*(*x*, *t*) be the mass flux density giving the amount of species that, at the time *t*, crosses (in the direction of the *x*-axis) the orthogonal unit-area section at the location *x* in the unit of time. The amount of chemical species released from the interface up to the time $t_{\mathrm{tot}}$ is then given by

1 $$\begin{aligned} m_{\mathrm{ext}} = - A \int _0^{t_{\mathrm{tot}}} dt \, J(x=0,t) \end{aligned}$$

For diffusive transport, *J*(*x*, *t*) is expressed by the first Fick’s law

2 $$\begin{aligned} J(x,t) = -D \frac{\partial c(x,t)}{\partial x} \end{aligned}$$

and the evolution of *c*(*x*, *t*) is ruled by the one-dimensional diffusion equation (second Fick’s law)

3 $$\begin{aligned} \frac{\partial c(x,t)}{\partial t} =-\frac{\partial J(x,t)}{\partial x} \end{aligned}$$

Such equation is complemented by the initial condition $c(x,0)=c_0$ (initial homogeneity at equilibrium) and by the specific boundary conditions corresponding to release or pause phases. During the release phases there is leakage of the species through the interface, while during the pause phases the interface behaves as a reflecting boundary and the concentration partially relaxes back to the flat profile (which would be attained after an infinitely long pause). Mathematically, this corresponds to set $c(x=0,t) = 0$ during the release phases (absorbing boundary at the interface), and $J(x=0, t)=0$ during the pause phases (reflecting boundary). In addition, for a material with infinite thickness one has that $\displaystyle {\lim _{x \rightarrow \infty }} J(x,t) = 0$ (and $\displaystyle {\lim _{x \rightarrow \infty }} c(x,t) = c_0$) corresponding to the fact that the conditions infinitely far from the interface are stationary and unaffected by the leakage of species.

The calculation of $m_{\mathrm{ext}}$ by means of Eq. (1) first requires the solution of Eq. (3) to compute the mass flux density from Eq. (2). The specific alternation release-pause makes that $m_{\mathrm{ext}}$ depends on the given operation schedule. In the general case of multiple release-pause phases, the solution of Eq. (3) can be obtained only numerically. The method used here is based on the basic finite-difference scheme outlined in “Numerical solution of the diffusion equation” Section.

As said above, the interest here is not in the numerical solution of the problem given a specific schedule; rather, our purpose is to work out bounds/approximations on $m_{\mathrm{ext}}$ getting rid of the schedule details. This calls for a global statistical synthesis of the specific schedule-dependent outcomes. The results are presented in the next section.

## Results

### Infinite thickness

In the limit of infinite thickness, the diffusion equation has an analytical solution only for a single release phase. Namely, the well-known solution (see for instance ref.^13^) is $c(x,t) = c_0 \, \mathrm{erf}(x/\sqrt{4 D t})$ with $\mathrm{erf}(\cdot )$ the Error Function defined as $\mathrm{erf}(u) = (2/\sqrt{\pi }) \int _0^u du' e^{-u'^2}$ for argument $u \ge 0$. In this case, the amount of species released up to time $t_{\mathrm{tot}}$ is

4 $$\begin{aligned} \mathrm{For} \;\; N =1 \;\; : \;\; m_{\mathrm{ext}} = {K \, \sqrt{t_{\mathrm{tot}}}} \end{aligned}$$

where

5 $$\begin{aligned} K = 2 \, A \, c_0 \sqrt{D / \pi } \end{aligned}$$

**Figure 2:**
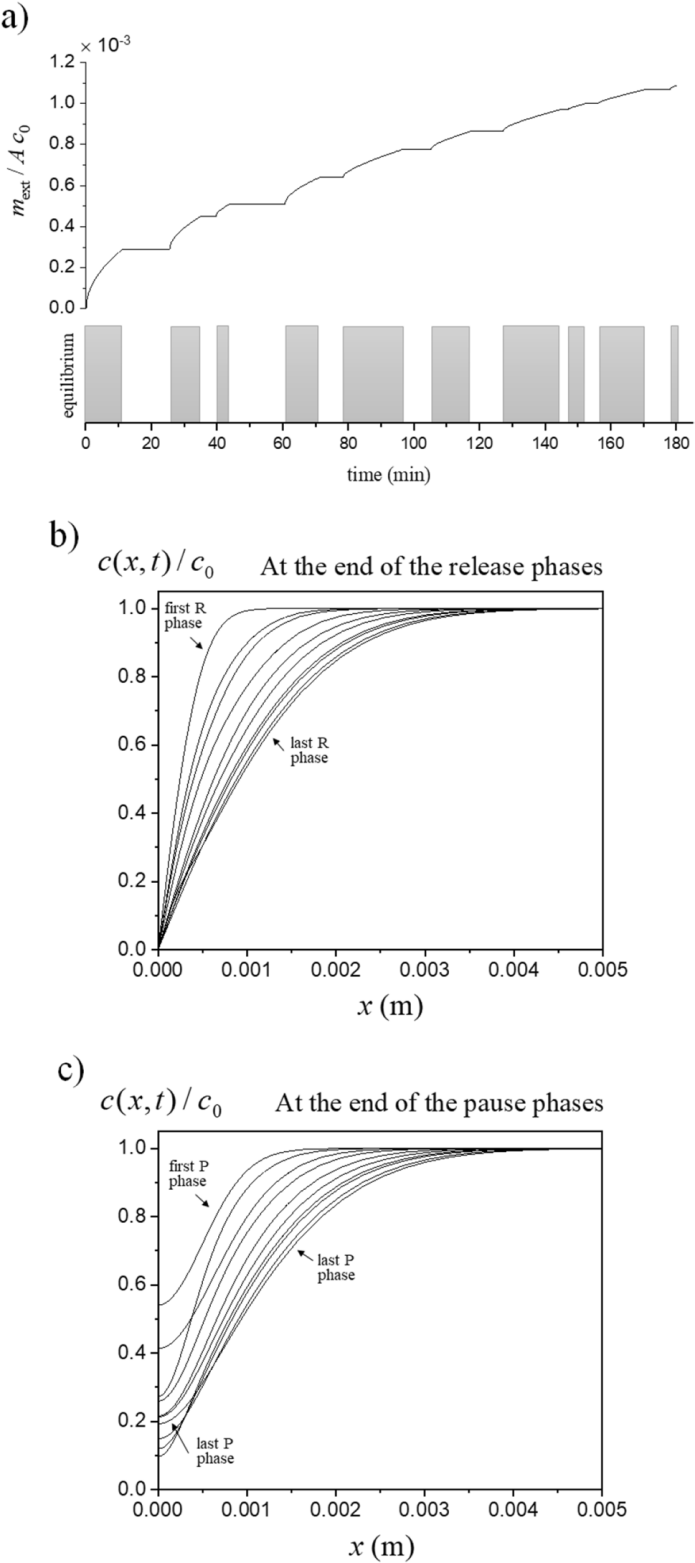
A sequence of release-pause phases with times randomly generated under the constraint $t_{\mathrm{tot}} = 3$ h. In this example, $N = 10$, $D = 10^{-10} \mathrm{m^{2} /s}$, and the material is supposed of infinite thickness. In panel (**a**), the shadowed blocks represent the release phases and the profile on the top shows the evolution of released quantity (here divided by $A \, c_0$ yielding a quantity expressed in meters). Panels (**b** and **c**) show, respectively, the concentration profiles at the end of the release and pause phases.

In the general case of multiple release-pause phases, the solution has to be obtained numerically, for instance by means of a finite-difference method (see “Methodological aspects and analysis” Section). This is because the concentration profile inside the material partially relaxes during the pause phases, hence a history-dependent initial condition has to be considered at the beginning of each new release phase. An example is shown in Fig. 2 for $N = 10$, in which the duration of each release and pause phase was randomly generated under the requisite that $t_{\mathrm{tot}} = 3$ h; the diffusion coefficient was set equal to $D = 10^{-10} \mathrm{m^{2} /s}$, which is typical of molecular diffusion in viscous stagnant liquids or liquid-like phases (e.g., gels). The curve on the top of panel a) shows the evolution of $m_{\mathrm{ext}}$. The dependence on the details of the operation schedule makes that what one can work out are only lower and upper *bounds* on $m_{\mathrm{ext}}$, or, at best, a good estimate.

An a priori lower bound can be established by noting that, at given total release time, $m_{\mathrm{ext}}$ is surely higher than the quantity released if there were a single release phase (Eq. 4). This is because the relaxation during the pause phases implies some flattening of the concentration profile with partial return back to the initial condition, hence a consequent higher slope at the interface when the release starts again. Thus, one expects that

6 $$\begin{aligned} m_{\mathrm{ext}} > K \, \sqrt{N} \, \sqrt{\overline{\tau }} \end{aligned}$$

where we have introduced the average time of a release phase, $\overline{\tau }$, defined as

7 $$\begin{aligned} \overline{\tau } = \frac{1}{N} \sum _{k=1}^N \tau ^{\mathrm{R}}_k \end{aligned}$$

On the opposite side, $m_{\mathrm{ext}}$ is surely lower than the quantity released when the pause phases are infinitely long, so that the initial condition is completely restored when the new release phase begins. In this way, the slope of the concentration profile at the interface is always the highest, so yielding the highest leakage rate. Thus, one expects that

8 $$\begin{aligned} m_{\mathrm{ext}} < K \sum _{k=1}^N \sqrt{\tau _k^{\mathrm{R}}} \le K \, N \, \sqrt{\overline{\tau }} \end{aligned}$$

where the last inequality follows from the application of Jensen’s inequality. For a concave function *f*(*x*) (i.e., a function with negative second derivative), such inequality states that $\langle {f (x)} \rangle \le f( \langle x \rangle )$ where the averages are meant to be taken over a generic distribution on the *x* variable. The inequality is here applied to the concave function square-root, and the average is the arithmetic mean over the *N* phases. Namely, $N^{-1} \sum _k \sqrt{\tau _k^{\mathrm{R}}} \le \sqrt{N^{-1} \sum _k \tau _k^{\mathrm{R}}} = \sqrt{\overline{\tau }}$ from which Eq. (8) readily follows. The a priori lower and upper bounds given above have been validated by numerical simulations (see below). Note that, for $N=1$, the terms in Eqs. (6) and (8) become equal one each other, and equivalent to the exact solution as well. Unfortunately, the upper bound in Eq. (8) becomes rapidly more and more loose as *N* increases.

As detailed in “Methodological aspects and analysis” Section, we also get an empirical approximation of $m_{\mathrm{ext}}$ by combining the numerical solution of the diffusion equation inside the material with a heuristic analysis of the outcomes from a large number of simulated instances. This is our main achievement. The result is (see “Methodological aspects and analysis” Section for details):

9 $$\begin{aligned} m_{\mathrm{ext}} \; \simeq \frac{K \, N \, \sqrt{\overline{\tau }}}{1 + \left( \sqrt{N} - 1\right) f(z)} \end{aligned}$$

where *f*(*z*) is the empirical function

10 $$\begin{aligned} f(z) = \left\{ \begin{array}{c} \displaystyle {0.2 + \frac{0.8}{1 + 0.6 \, z}} \;\; \mathrm{for} \; 0 \le z \le 10 \\ \\ 0 \;\; \mathrm{for} \; z > 10 \end{array}\right. \end{aligned}$$

whose dimensionless argument *z* is the ratio between the average time of a pause phase and the average time of a release phase; in terms of $t_{\mathrm{tot}}$, $\overline{\tau }$ and *N*, it reads

11 $$\begin{aligned} z = \frac{t_{\mathrm{tot}} - N \overline{\tau }}{(N-1) \overline{\tau }} \end{aligned}$$

With respect to Eq. (4), Eq. (9) may be seen as a adjusting expression for $m_{\mathrm{ext}}$ giving a plausible estimate when $N > 1$. The geometrical parameters and the diffusion coefficient still enter only through the factor *K*, while the dependence on the schedule enters through *N* and the function *f*(*z*). The crucial variable related with the essential features of the time schedule is *z*. As the cumulative duration of the pauses tends to a low fraction of $t_{\mathrm{tot}}$, the value of *z* tends to zero; in contrast, for short cumulative duration of the release phases, *z* takes values more and more high. Since $0 \le f(z) \le 1$, the value of the right-hand side Eq. (9) is always comprised between the two bounds Eqs. (6) and (8). The lower bound is approached from above as $z \rightarrow 0$ where $f(z) \rightarrow 1$ (and, in practice, the lower bound reduces to Eq. (4) as if there were a unique release phase since the pauses are extremely short and can be neglected). On the contrary, as $z \rightarrow \infty$, *f*(*z*) is null and Eq. (9) approaches the highest bound Eq. (8) (which, in practice, tends to become vanishingly small as if there were a unique pause phase without release of the species). The fact that the diffusion coefficient enters only the multiplicative factor *K* can be expected on the basis of scaling arguments by considering that in the infinite-thickness case, in the absence of any characteristic length, a variation of *D* does simply reflect on a homogeneous scaling of the dynamics. Accordingly, it is not surprising that the schedule-dependent adjustment is expressed in terms of a ratio between characteristic times of the schedule itself. On the other hand, the dependence exactly on *z* (ratio between the average time of pause and release) and the specific form of *f*(*z*) are non-trivial features that derive from the heuristic inspection.

A comment is due about the “$\simeq$” in Eq. (9). Following the inspection in “Methodological aspects and analysis” Section it is seen that Eq. (9) should be an upper bound to $m_{\mathrm{ext}}$ more stringent than Eq. (8). Such a refined bound, however, is not mathematically derived nor strict; rather, its violations are admitted but are found to be little as shown below. In practice, Eq. (9) proves to provide a good estimate (much probably a slight overestimate) of $m_{\mathrm{ext}}$. For finite *z* values, we directly assessed (see below) the effectiveness of Eq. (9) by generating ensembles of instances and looking at (1) the collocation of the exact values of $m_{\mathrm{ext}}$ with respect to quantity expressed by Eq. (9), and (2) the distribution of the relative deviation of $m_{\mathrm{ext}}$ from such approximation.

Concerning the status of Eq. (9), we stress again that it constitutes an empirical achievement based on an heuristic inspection. In this sense, it has to be taken as a “discovery” from numerical experiments conducted in unbiased way, and then subjected to a proof of effectiveness. The heuristic inspection fills the gap between exact formal mathematical treatment (which, as said above, is hampered by the variety of schedule-dependent initial conditions at the beginning of each release phase) and plausible estimate of $m_{\mathrm{ext}}$ given the essential features of the schedule. The price to pay is the loss of direct connection between Eqs. (9) – (11) and the physical features of the diffusion/release process. Of course, as for any empirical result, improvements of Eq. (9) (either in terms of functional form and/or values of the parameters) could be achieved by accumulating and analyzing a larger set of simulations. We also stress the crucial fact that Eq. (9) is strictly applicable only to the case of release under diffusion from materials of infinite thickness and planar interfaces, and for initial uniform load of the species; it is expected that if any of such assumptions is relaxed, then Eq. (9) needs to be replaced by a different relation to be discovered case by case by means of the same heuristic approach. Notably, the numerical parameters entering Eq. (10) are intrinsic dimensionless coefficients characteristic of the physical setup but independent of the details of the specific case; in this lies the utility of Eq. (9).

**Figure 3:**
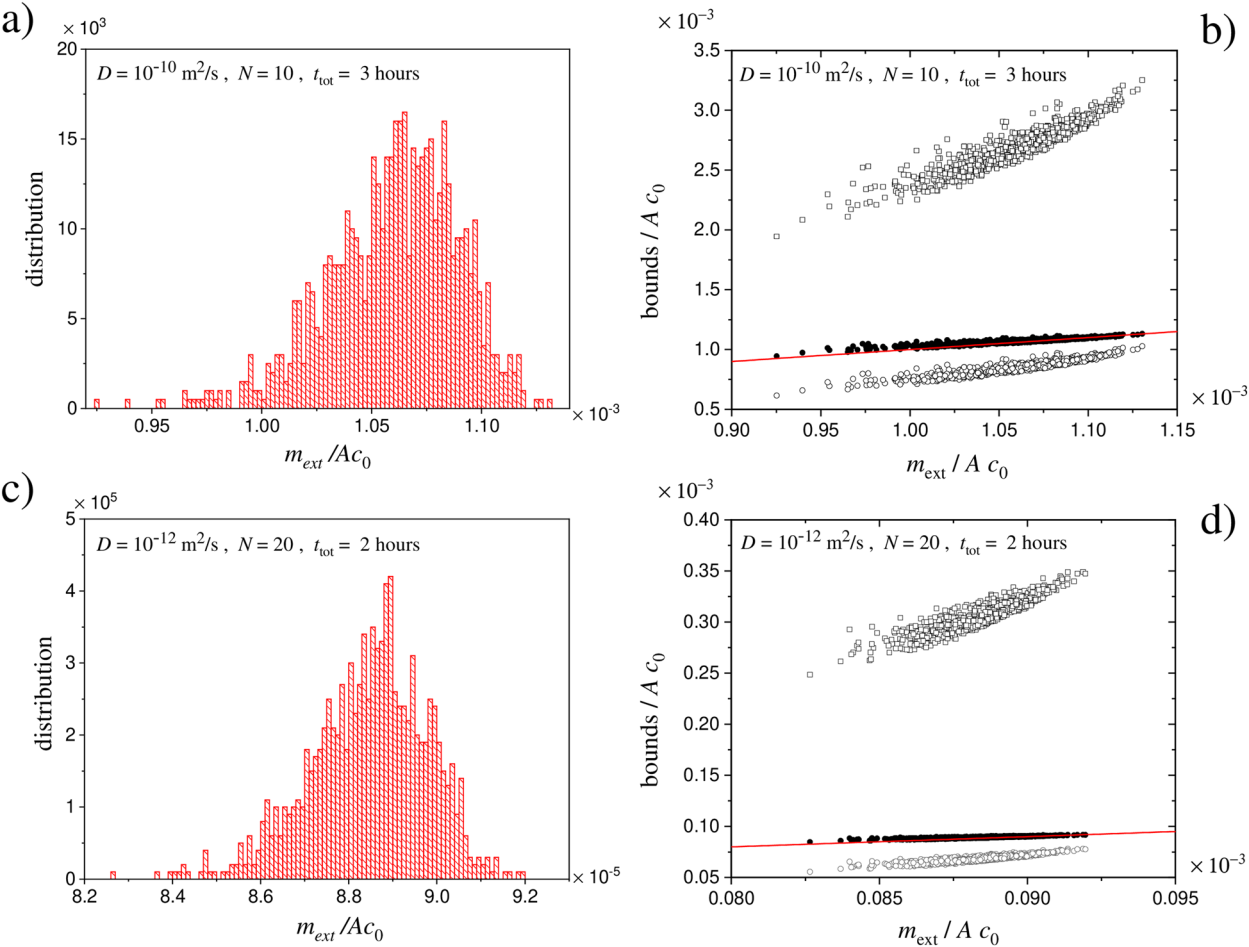
Panels (**a** and **c**) show examples of the distribution of the released quantity $m_{\mathrm{ext}}$, for a material of infinite thickness, as the release and pause times are randomly generated at fixed *N* and total time $t_{\mathrm{tot}}$. For the same instances of panels (**a** and** c**), panels (**b** and** d**) show the interrelation between the exact value of $m_{\mathrm{ext}}$ and the lower bound in Eq. (6) (open circles), the upper bound in Eq. (8) (open squares), and the expression in Eq. (9) (filled circles). The continuous red line has slope one. Note that the released quantities are divided by $A \, c_0$, hence have physical dimension of a length (here expressed in meters).

**Figure 4:**
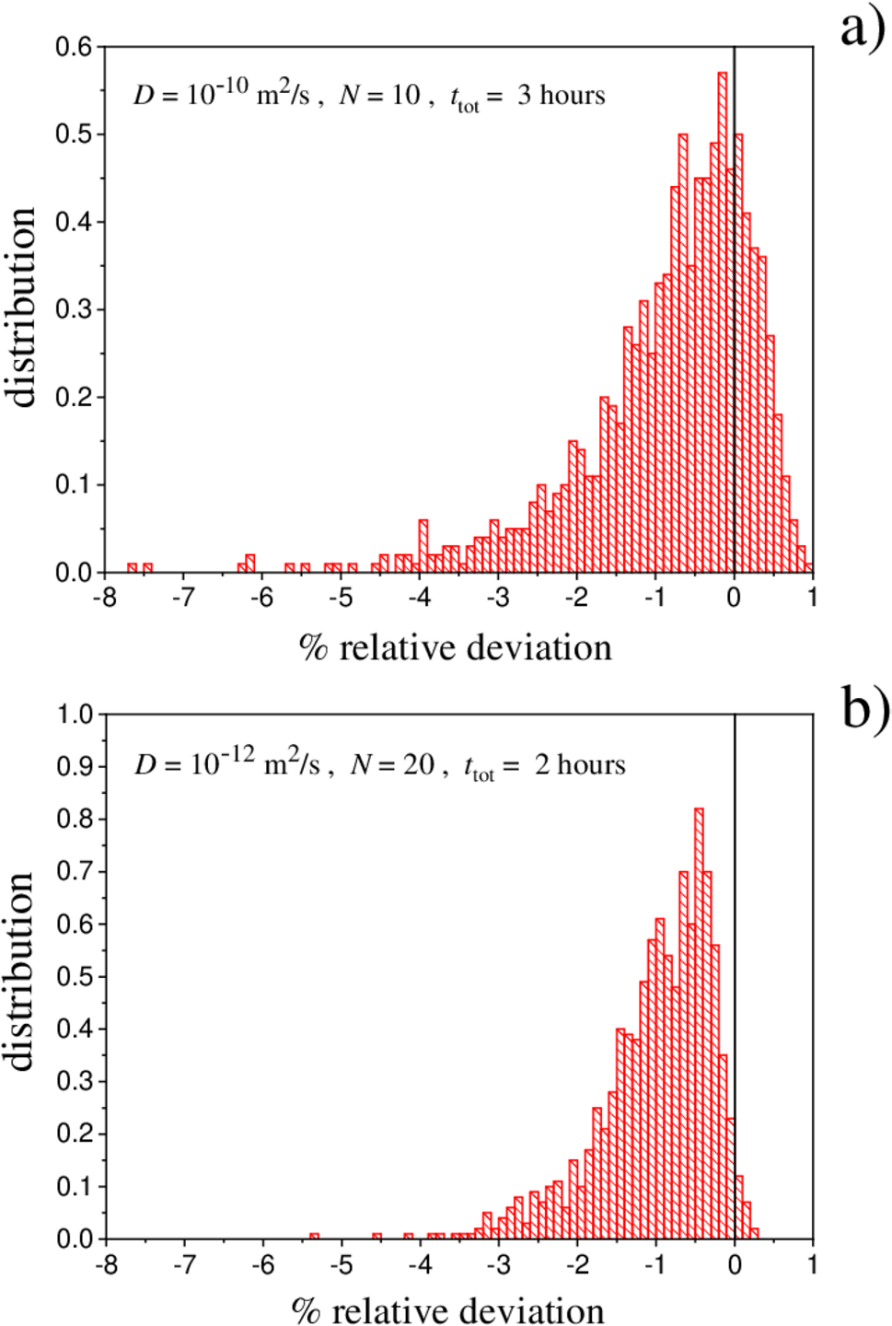
Example of distribution of the percentage relative displacement of $m_{\mathrm{ext}}$ from $m_{\mathrm{approx}}$ for a material of infinite thickness. The abscissa values are $100 \times (m_{\mathrm{ext}} - m_{\mathrm{approx}})/m_{\mathrm{ext}}$, where $m_{\mathrm{approx}}$ has been calculated with Eq. (9). The conditions are the same of Fig. 3.

As illustrative examples we considered here two cases: (1) $D = 10^{-10} \, \mathrm{m^2 /s}$, $N=10$, $t_{\mathrm{tot}} = 3$ h, and (2) $D = 10^{-12} \, \mathrm{m^2 /s}$, $N=20$, $t_{\mathrm{tot}} = 2$ h. While case (1) pertains the release from liquid-like materials (e.g., gels), in case (2) the value of *D* is closer to the typical values in plastic materials^6,8^. Just to make an example in the dermal exposure context, case (2) could be the leakage of some chemical species from a highlighter handled twenty times in the course of 2 h of office work. At fixed total time, 1000 instances have been generated by randomly drawing in unbiased way the duration of the release/pause phases. For each generated time-schedule, the exact value (under the required accuracy criteria) of $m_{\mathrm{ext}}/A \, c_0$ has been computed solving the diffusion equation by means of the finite-difference scheme described in “Methodological aspects and analysis” Section. Figure 3 shows the outcomes. Panels (a) and (c) show the distribution of the released quantity. This gives an indication about the spread of the values if the duration of the release and pause phases is generated at random at fixed *N* and $t_{\mathrm{tot}}$. For the same data sets, panels (b) and (d) show the lower bound Eq. (6) (open circles), the upper bound Eq. (8) (open squares) and the approximation Eq. (9) (filled circles) versus the exact values; the continuous red line has slope one. The figure reveals the closeness of the approximation to the true value. The fact that Eq. (9) mostly gives a slight overestimation (but well below the a priori bound in Eq. (8)) is illustrated in Fig. 4 showing the statistical distribution of the percentage relative deviation $100 \times (m_{\mathrm{ext}} - m_{\mathrm{approx}})/m_{\mathrm{ext}}$, where $m_{\mathrm{approx}}$ is the value at the right-hand side of Eq. (9). Such a relative deviation serves to quantify both the violation of the bound in Eq. (9) (occurrence of positive values) and the closeness of $m_{\mathrm{approx}}$ to the true value. The percentage of instances in which $m_{\mathrm{ext}}$ exceeds $m_{\mathrm{approx}}$ was found to depend on the parameters employed. The percentage was of $23 \%$ for case (1) and of $2 \%$ for case (2). In both cases, however, such violations correspond to values confined within 1% above the approximation.

In summary, the true values of $m_{\mathrm{ext}}$ are highly concentrated around the right-hand side of Eq. (9), meaning that such an empirical expression gives a good estimation of $m_{\mathrm{ext}}$ (generally a slight overestimation) by avoiding cumbersome exact calculations which would require the details of the time schedule of the release/pause phases.

### Finite-thickness correction

On the basis of the heuristic analysis illustrated in “Finite-thickness analysis” Section, we can make an adjustment of the approximation Eq. (9) when the finiteness of the material’s thickness, *b*, cannot be ignored. Given the release-pause schedule, it is found that the key control parameter is the dimensionless quantity

12 $$\begin{aligned} \sigma =\frac{b}{\sqrt{2 D \, t^{\mathrm{R}}_{\mathrm{tot}}}} \end{aligned}$$

where $t^{\mathrm{R}}_{\mathrm{tot}}$ is the total release time (hence $t^{\mathrm{R}}_{\mathrm{tot}} = N \, \overline{\tau }$). Letting $m_{\mathrm{ext}, \infty }$ be the amount of species released from the material as if it had an infinite thickness, for the true amount $m_{\mathrm{ext}}$ it is found that

13 $$\begin{aligned} m_{\mathrm{ext}} \le m_{\mathrm{ext},\infty } \; u(\sigma ) \end{aligned}$$

with the upper-bound function

14 $$\begin{aligned} u(\sigma ) = \left\{ \begin{array}{l} \sigma \sqrt{\pi /2} \;\; \mathrm{for} \;\; \sigma \le 0.8 \\ 1 \;\; \mathrm{for} \;\; \sigma > 0.8 \end{array}\right. \end{aligned}$$

where 0.8 is the approximation of $\sqrt{2/\pi }$. As commented in “Finite-thickness analysis” Section, a tentative lower bound on $m_{\mathrm{ext}}$ could also be given, but further numerical investigations are required to make sound statements.

Equation (13), in combination with Eq. (9), allows us to provide an upper bound on the released quantity taking into account the finiteness of the thickness. Let us introduce the thickness-dependent characteristic time

15 $$\begin{aligned} t^*= \frac{\pi \, b^2}{4 D} \end{aligned}$$

For $t^{\mathrm{R}}_{\mathrm{tot}} > t^*$, from Eq. (13) with Eq. (9) we get $m_{\mathrm{ext}} \le {c_0 \, A \, b \, \sqrt{N}}/[1 +(\sqrt{N} - 1) f(z)]$ where the “$\le$” is here likely applicable by considering the “$\simeq$” of Eq. (9) combined with the “$\le$” of Eq. (13). This inequality is however trivial. In fact, $c_0 \, A \, b$ corresponds to the total quantity of species that is present in the portion of slab material, and $\sqrt{N}/[1 +(\sqrt{N} - 1) f(z)] \ge 1$ because *f*(*z*) is comprised between 0 and 1; thus the inequality states nothing but that $m_{\mathrm{ext}}$ cannot exceed the total amount of species initially present in the material. On the other side, for $t^{\mathrm{R}}_{\mathrm{tot}} \le t^*$, we get the following inequality (corresponding to Eq. (9)) which is potentially useful:

16 $$\begin{aligned} m_{\mathrm{ext}} \le \frac{2 \, c_0 \, A \, \sqrt{D \overline{\tau }/\pi } \, N}{1 +(\sqrt{N} - 1) f(z)} \;\;\, \mathrm{for} \;\; t^{\mathrm{R}}_{\mathrm{tot}} \le t^*\end{aligned}$$

While Eq. (16) is mathematically always fulfilled, it becomes non-trivial only if $t^{\mathrm{R}}_{\mathrm{tot}}$ is sufficiently smaller than $t^*$. Specifically, the right-hand side of Eq. (16) turns out to be smaller than the initial total amount of species in the portion of material (and hence the inequality is non-trivial) only if

17 $$\begin{aligned} t^{\mathrm{R}}_{\mathrm{tot}} \le t^*\, \gamma (N, \overline{\tau }, t_{\mathrm{tot}}) \;\;\; , \;\;\; \gamma (N, \overline{\tau }, t_{\mathrm{tot}}) =\frac{[1 +(\sqrt{N} - 1) f(z)]^2}{N} \end{aligned}$$

The factor $\gamma (N, \overline{\tau }, t_{\mathrm{tot}})$ lies between 1/*N* and 1, hence the condition of Eq. (17) is more stringent than $t^{\mathrm{R}}_{\mathrm{tot}} \le t^*$. If Eq. (17) results to be violated, Eq. (16) is still valid but useless since we could only state that $m_{\mathrm{ext}}$ does not exceed the total quantity of species initially present in the portion of material. In essence, the specific release-pause schedule controls the effectiveness of Eq. (16). A numerical exemplification will be provided in  “Examples” Section.

As $t^{\mathrm{R}}_{\mathrm{tot}}$ decreases, Eq. (16) becomes more and more stringent up to reach its full effectiveness when $t^{\mathrm{R}}_{\mathrm{tot}}$ is enough short that the material would behave, in practice, as if its thickness were infinite. Further inspections are however needed to quantitatively characterize the transition to the infinite-thickess-like situation.

As a final remark, we underline that although Eq. (16) is referred to as a formula for the finite-thickness correction, the thickness *b* does not enter explicitly. Rather, *b* enters indirectly through $t^*$, that is, in the specification of the range of applicability of Eq. (16). In other words, without providing the value of *b*, Eq. (16) would be meaningless.

### Examples

Let us make an example to clarify the application of the results presented in “Finite-thickness correction” Section. In particular, we want to highlight how the factor $\gamma (N, \overline{\tau }, t_{\mathrm{tot}})$, which depends on the schedule of release-pause phases, determines the effectiveness of the bound in Eq. (16).

Suppose to deal with the release of a species whose diffusion coefficient is $D = 10^{-13} \, \mathrm{m^2/s}$ in a plastic material of thickness $b = 0.1 \, \mathrm{mm}$. The characteristic time $t^*$ is about 22 h. Given the value of $t^*$, which is an intrinsic parameter, the chemical’s release depends of the specific schedule of release (contact)-pause phases. Let us now consider three situations.

- The material is touched $N = 10$ times with total contact time $t^{\mathrm{R}}_{\mathrm{tot}} = 5$ h, and with $t_{\mathrm{tot}} = 10$ h (including the pauses between the contacts, see Fig. 1). With such a schedule we get $z = 1.11$ and $f(z)=0.68$. From Eq. (17) we get $\gamma (N, \overline{\tau }, t_{\mathrm{tot}}) = 0.61$ and $t^*\, \gamma (N, \overline{\tau }, t_{\mathrm{tot}}) = 13.3$ h. In such a case, the condition in Eq. (17) is fulfilled and Eq. (16) sets a non-trivial bound: $m_{\mathrm{ext}} \le 0.61 \, c_0 \, A \, b$.
- Consider $N = 30$ contacts with total contact time $t^{\mathrm{R}}_{\mathrm{tot}} = 15$ h and $t_{\mathrm{tot}} = 30$ h. In this case, $z=1.03$, $f(z)=0.69$. This gives $\gamma (N, \overline{\tau }, t_{\mathrm{tot}}) = 0.56$ and $t^*\, \gamma (N, \overline{\tau }, t_{\mathrm{tot}}) = 12.3$ hours. The condition of Eq. (17) is violated and Eq. (16) becomes trivial. In fact, we would get $m_{\mathrm{ext}} \le 1.106 \, c_0 \, A \, b$, which is obvious a priori because $c_0 \, A \, b$ is the total amount of species initially present in the portion of material under consideration. The only sound, but obvious, statement that we can make is that $m_{\mathrm{ext}}$ is at most equal to $c_0 \, A \, b$.
- Suppose to be in the situation of very large number of contacts with short total contact time, say $N = 100$ and $t^{\mathrm{R}}_{\mathrm{tot}} = 15$ minutes, and with long total pause time giving $t_{\mathrm{tot}} = 10$ hours. With such parameters we have $z = 39.4$ and hence $f(z) = 0$ according to the clause in Eq. (10). Thus, $\gamma (N, \overline{\tau }, t_{\mathrm{tot}}) = 1/N = 10^{-2}$, which leads to $t^*\gamma (N, \overline{\tau }, t_{\mathrm{tot}}) = 13.3$ minutes slightly shorter than $t^{\mathrm{R}}_{\mathrm{tot}}$. Since Eq. (17) is violated, what we can state is simply that $m_{\mathrm{ext}}$ cannot exceed $c_0 \, A \, b$. On the other hand, at fixed *N* and $t^{\mathrm{R}}_{\mathrm{tot}}$ we find that *z* goes above 10 when $t_{\mathrm{tot}}$ crosses a value between 2.5 and 3 hours; this is associated with an abrupt change of the factor $\gamma (N, \overline{\tau }, t_{\mathrm{tot}})$ which, in this case, makes that Eq. (16) suddenly turns from a useful bound ($m_{\mathrm{ext}} \le 0.27 \, c_0 \, A \, b$ for $t_{\mathrm{tot}} = 2.5$ hours) to the trivial bound ($m_{\mathrm{ext}} \le c_0 \, A \, b$ for $t_{\mathrm{tot}} = 3$ hours). This example serves to highlight that the poor characterization of the tail of the function *f*(*z*) for $z > 0$ may cause the sudden switch from a relevant bound to the safe but trivial upper bound. We recall that large values of *z* are realized for large ratios between average duration of the pause phases and average duration of the release phases, as it is in the present example.

## Remarks and perspectives

In this work we have dealt with the intermittent release of chemical species from materials. We have assumed uniform initial load, diffusive transport in the material, and irreversible escape of the chemicals through the interface taken as planar. The statistical synthesis of simulated release experiments allowed us to provide a simple empirical expression to estimate (slightly overestimate) the quantity of chemical species released through the interface of a material of infinite thickness. A finite-thickness correction was then implemented. Despite its simplicity, the physical setup adopted here constitutes the basis for the tier modeling of dermal exposure^6^. Here we have added the effect of the release-pause alternation, which is important if the chemicals are released to the skin from tools or consumer objects when they are used/touched in discontinuous way, as it normally happens in practice. Apart from such application, the methodology and the results are however applicable to any other situation which conforms to the assumptions made. The long-term potential applications are manifold, including ambits like environmental and regulatory toxicology in which is important to quantify the leakage of chemicals from a hosting phase to other phases (air, water, tissues, etc.).

To the best of our knowledge, semi-empirical results like those in Eqs. (9) (infinite thickness) and (16) (finite thickness) are a novelty in the field of release of chemicals from planar interfaces under diffusion with initial uniform load of the species. We stress again that the novelty does not lie in the diffusive model (an ambit largely inspected in past works), but in the idea of extracting likely approximations of the released quantity from the analysis of a set of simulated instances keeping fixed the parameters that specify the release-pause schedule. On the methodological side, a due further work is to make a detailed inspection about the range of validity of the results, improve them, and possibly relax some of the assumptions of the model. In particular, the characteristic function *f*(*z*) defined in Eq. (10) is currently not well characterized for $z > 10$, i.e., in cases when the average duration of the pause phases is more than 10 times the average duration of the release phases; in turn, when *z* is above 10, the estimate of $m_{\mathrm{ext}}$ abruptly switches to the safe but large a priori bound. To achieve a better characterization of the tail of *f*(*z*) at large *z* it is required to run a very extended set of simulations like those described in “Heuristic construction of Eq. (9)” Section, in order to explore a wider set of parameters compatible with values $z > 10$. We are also going to carry out experiments with model setups in order to check the effectiveness of the approximations presented here by realizing the practical equivalent of the numerical simulations; this will be the content of a forthcoming publication.

Several lines of development can be drawn. First, it would be interesting to account for the possibility of crossing the interface from both sides, that is to include, among the parameters, also the thermodynamic partition constant of the chemical species between material and exterior. In addition, also the geometry of the material should be taken into account for a realistic description of the chemical’s release in timescales long enough. Analytical solutions of the diffusion equation are available for various geometries and uniform initial condition (see for instance refs.^1,10^ and^13^) but, again, the effect to the release-pause alternation has not been inspected yet. Although non-planar geometries might be of little relevance in the dermal exposure context mentioned above, their study would be useful for understanding to what extent Eq. (9) is affected by the shape as the dimension of the object is ever decreased. In this regard, it would be interesting to consider the spherical geometry for which analytical models are available^11,13^ and currently applied to the release of chemicals from plastic microparticles (see for instance ref.^14^). An intermittent release would be here hardly conceivable in practice but, as stated, the application of the same heuristic analysis made for the planar case could possibly lead to a different empirical approximation of $m_{\mathrm{ext}}$; the comparison with Eq. (9) should give an indication about the sensitivity with respect to the geometrical features. A further line of extension is to consider non-uniform initial loads of the species inside the material. Again, this would lead to different results case by case. In summary, what we have considered here is only one setup among many others, in our opinion the most relevant one for an initial inspection.

Still on the methodological side, we claim that an heuristic approach like the one presented here, based on the statistical analysis of simulated instances, might provide useful empirical bounds on the chemical release even for more complex setups in which the heterogeneity of the material plays a role, the dynamics are no more diffusive, and the external medium penetrates in the material changing its properties. Just to mention, in the context of controlled drug release from medical devices it is known that the release is in principle affected by many processes other than diffusion (e.g., matrix swelling and erosion, osmotic effects, diffusion of water in the device, drug dissolution if the drug is dispersed in the matrix, etc.)^10,15^. Despite the complexity of such systems, in many situations the effective release during a single release phase can be described, at least in a limited time window to be assessed case by case, by simple power-law relations of the kind $m_{\mathrm{ext}} \propto t^n$ where *n* is a characteristic exponent^16^; for instance, $n = 1/2$ for the release from a slab geometry either in case the species is dissolved in the matrix or dispersed forming a separate phase (according to the celebrated Higuchi equation ^17,18^). What emerges from our study is that the release-pause alternation might add a further dimension to the control of the chemical release. In this perspective, it would be interesting to apply our kind of heuristic approach, case by case, also to such situations.

## Methodological aspects and analysis

### Numerical solution of the diffusion equation

The numerical solution of Eq. (3) has been obtained by employing a finite-difference scheme with non-uniform partition of the *x*-axis truncated at a given depth *L* from the interface. At *L*, a reflecting boundary is imposed. If the material is meant to have infinite thickness, *L* is taken large enough to ensure that such a truncation does not introduce artifacts. If the material has a finite thickness *b*, then $L = b$ is applied.

Specifically, in the case of infinite thickness we have employed a thinner homogeneous discretization up to the depth $L_{\mathrm{turn}} =\sqrt{2 D t_{\mathrm{tot}}}$ (corresponding to the root mean squared displacement of a molecule orthogonally to the interface in the time $t_{\mathrm{tot}}$), and then a homogeneous discretization with wider intervals from $L_{\mathrm{turn}}$ to $L= \gamma \, L_{\mathrm{turn}}$ where $\gamma$ is a multiplicative factor to be fixed. The number of intervals in the two sectors, and the multiplicative factor $\gamma$, were enlarged up to reach a prescribed convergence on the numerical outcomes. Specifically, with reference to the infinite-thickess case we have implemented two criteria to establish if convergence was reached: (1) the concentration *c*(*L*, *t*) must remain close to $c_0$ within a certain percentage tolerance $\varepsilon _1$ up to the time $t_{\mathrm{tot}}$, and (2) the numerical value of $J(x=0,t)$ must remain close, within a percentage tolerance $\varepsilon _2$, to the value from the analytical solution in the case of a unique release phase of duration $t_{\mathrm{tot}}$. In all calculations performed in this work, $\gamma = 50$ was sufficient to satisfy such requisites. For the generation of the profiles in Fig. 2, a thin discretization with $N_1 = 700$ and $N_2 =300$ was employed to ensure convergence within $\varepsilon _1 = 2$ % and $\varepsilon _2 = 1$ %. For Figs. 3 and 4, $N_1 =200$ and $N_2 =100$ ensured convergence within $\varepsilon _1 = 2$ % and $\varepsilon _2 = 2$ %. For the heuristic determination of the approximation Eq. (9) (see below), $N_1 = 100$ and $N_2 =50$ were employed for $\varepsilon _1 = 5$ % and $\varepsilon _2 = 2$ %. In the case of finite thickness, if $b \le L_{\mathrm{turn}}$ the computation was carried out with a fixed number $N_1$ of intervals of width $b/N_1$. If $b > L_{\mathrm{turn}}$, the number of intervals within $L_{\mathrm{turn}}$ was set to $N_1$, and the number of intervals between $L_{\mathrm{turn}}$ and *b* was set equal to a fixed $N_2$. In the calculations made for producing Fig. 6 shown later, the employed numbers were $N_1 = 100$ and $N_2 = 100$ for panels from (a) to (d), while $N_1 = 100$ and $N_2 = 200$ for panel (e).

On computational grounds, the discretization of Eq. (3) leads to get the evolution law of the column vector $\tilde{\mathbf{c}}(t)$ whose components are $\tilde{c}_n(t) = c(x_n,t) \sqrt{\Delta _n}$ where $x_n$ is the central point of the *n*-th interval of width $\Delta _n$; the index *n* runs from 1 to $N_{\mathrm{int}}$, with $N_{\mathrm{int}}$ the total number of intervals into which the domain from $x=0$ to $x=L$ is partitioned. The reason for multiplying the concentration by the square root of the interval width is that, in such a way, the evolution matrix turns out to be symmetric, hence hermitian. Specifically, we get that $d \tilde{\mathbf{c}}(t)/dt =-\tilde{\mathbf{M}} \tilde{\mathbf{c}}(t)$ where the $N_{\mathrm{int}} \times N_{\mathrm{int}}$ matrix $\tilde{\mathbf{M}}$ can be either $\tilde{\mathbf{M}}^{\mathrm{R}}$ for the relaxation phases, or $\tilde{\mathbf{M}}^{\mathrm{P}}$ for the pause phases. Such matrices have a tri-diagonal form with diagonal elements $\tilde{M}^{\mathrm{R}}_{11} = \alpha ^{\mathrm{R}}_1$, $\tilde{M}^{\mathrm{P}}_{11} = \alpha ^{\mathrm{P}}_1$, $\tilde{M}^{\mathrm{R}}_{nn} = \tilde{M}^{\mathrm{P}}_{nn} =\alpha _n$ for $n=2, \ldots , N_{\mathrm{int}}$, and off-diagonal elements $\tilde{M}^{\mathrm{R}}_{n,n+1} (= \tilde{M}^{\mathrm{R}}_{n+1,n}) = \tilde{M}^{\mathrm{P}}_{n,n+1} (= \tilde{M}^{\mathrm{P}}_{n+1,n}) = \beta _n$ for $n=1, \ldots , N_{\mathrm{int}}-1$; in these expressions, $\alpha _n = 2 D \left[ (\Delta _n + \Delta _{n+1})^{-1} + (\Delta _{n-1} + \Delta _n)^{-1} \right] /\Delta _n$ for $2 \le n \le N_{\mathrm{int}}-1$, $\alpha _{N_{\mathrm{int}}} = 2 D ( \Delta _{N_{\mathrm{int}}-1} + \Delta _{N_{\mathrm{int}}} )^{-1}/\Delta _{N_{\mathrm{int}}}$, $\alpha ^{\mathrm{R}}_1 = 2 D \left[ (\Delta _1 + \Delta _2)^{-1} + (\Delta _0 + \Delta _1)^{-1} \right] /\Delta _1$, $\alpha ^{\mathrm{P}}_1 =2 D ( \Delta _1 + \Delta _2 )^{-1}/\Delta _1$, and $\beta _n = - 2 D ( \Delta _n + \Delta _{n+1} )^{-1} /\sqrt{\Delta _n \Delta _{n+1}}$. Here, $\Delta _0$ is the width of a fictitious interval at negative *x* values and opposed to the first interval; the choice $\Delta _0 \equiv \Delta _1$ was done. The explicit solution for $\tilde{\mathbf{c}}(t)$ during each release/pause phase initiating at a time $t_{\mathrm{init}}$ is given by $\tilde{\mathbf{c}}(t) = e^{-(t-t_{\mathrm{init}}) \tilde{\mathbf{M}}}\tilde{\mathbf{c}}(t_{\mathrm{init}})$ where the starting vector $\tilde{\mathbf{c}}(t_{\mathrm{init}})$ is meant to be determined by the solution of the previous part of the evolution starting from $\tilde{c}_n(0) = \sqrt{\Delta _n} \, c_0$ at the initial equilibrium. Numerically, such a calculation is performed through the decomposition of the exponential matrix on the basis of the eigenvectors of the matrix $\tilde{\mathbf{M}}$. Once $\tilde{\mathbf{c}}(t)$ has been determined in the whole time-window from 0 to $t_{\mathrm{tot}}$, we can compute $d m_{\mathrm{ext}}(t)/dt = - A \, J(x=0,t) \simeq A D c(x_1,t)/[(\Delta _0+\Delta _1)/2]$. The integration on *t* from 0 to $t_{\mathrm{tot}}$ finally yields the total amount $m_{\mathrm{ext}}$ released through the interface (note that such time integrations are analytical once the evolution kernels $e^{-(t-t_{\mathrm{init}}) \tilde{\mathbf{M}}}$ are decomposed on the eigenvectors basis; only the release phases do contribute). A FORTRAN code has been written for the calculations.

### Heuristic construction of Eq. (9)

For a given value of the diffusion coefficient *D*, of the number of release phases *N*, and of the average release time $\overline{\tau }$, we have performed a large number of simulations by drawing at random the duration of each release and pause phase and computing $m_{\mathrm{ext}}$. With such a setup, the total release time $t^{\mathrm{R}}_{\mathrm{tot}}$ is fixed to $N \overline{\tau }$. In the simulations we opted to use *D* and times expressed with physical units. This has been done in purpose because, for the infinite-thickness case, there is not an intrinsic scaling factor for the time variable, hence the use of dimensionless times built with subjectively chosen scaling factors would have introduced unnecessary complications in the specification of the conditions. It is implicit that simulations in which *D* is changed while all other parameters are kept fixed do not add new information (in the infinite-thickness case the dynamics are homogeneously slowed down or sped up), but they augment the set of outcomes at disposal for the post-production analysis.

**Figure 5:**
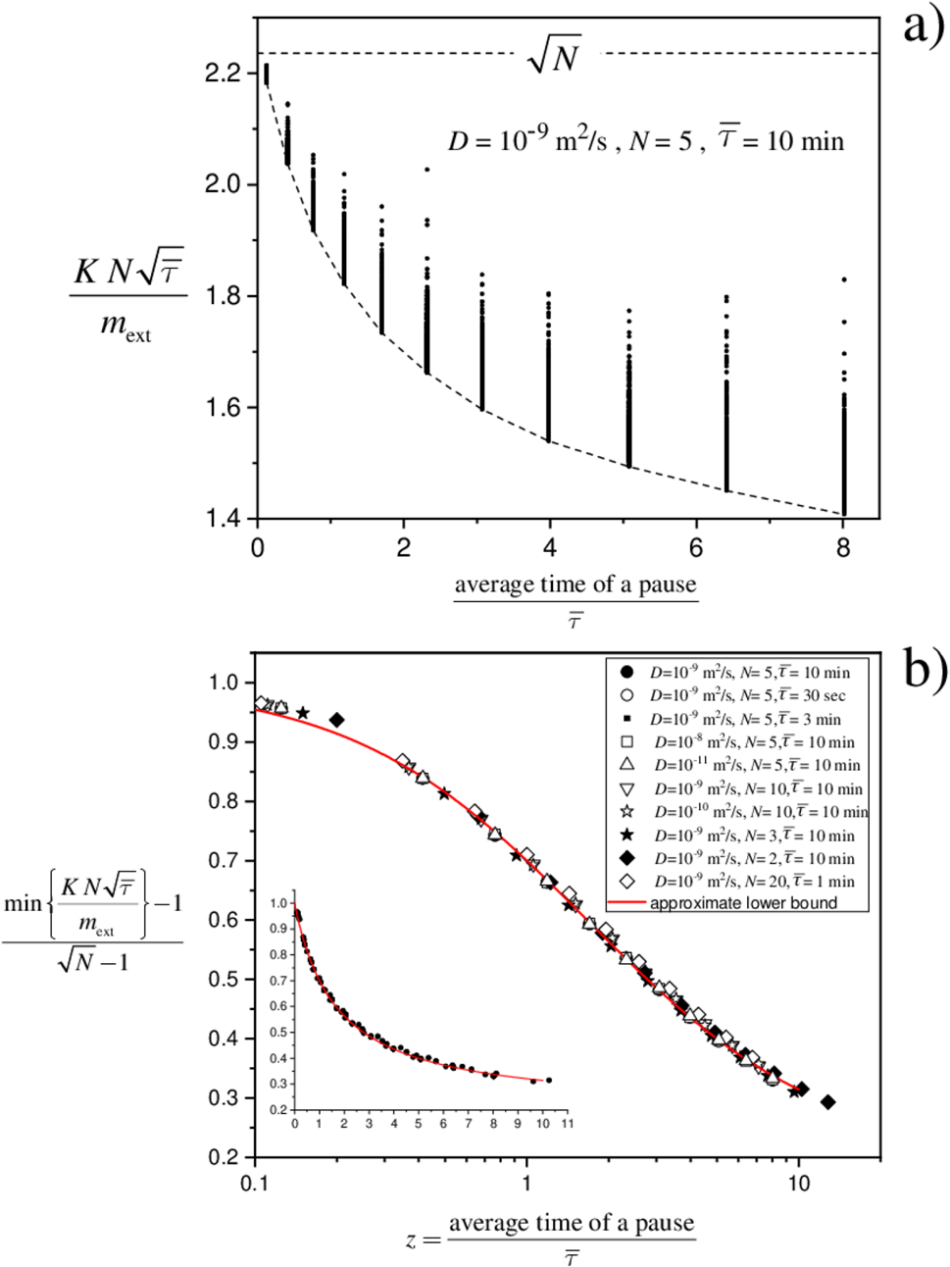
Heuristic basis of Eq. (9) for diffusion in a material of infinite thickness. Panel (**a**). Example of the spread of the factor $K \, N \, \sqrt{\overline{\tau }} / m_{\mathrm{ext}}$ for several ratios between average time of a pause phase and average time of a release phase. The plot reveals the existence of a lower bound. Panel (**b**). Rescaled plot of the lower bound obtained from data sets like that in Panel (**a**); each symbol refers to specific conditions (value of the diffusion coefficient *D*, number *N* of release phases, average time $\overline{\tau }$ of a release phase). The red curve is the empirical function *f*(*z*) in Eq. (10). The inset shows the same data with linear scale on the abscissa.

The total time $t_{\mathrm{tot}}$ has been varied between $1.1 \, t^{\mathrm{R}}_{\mathrm{tot}}$ (corresponding to short total time of pauses) and $50 \, t^{\mathrm{R}}_{\mathrm{tot}}$ (long pause time) taking 11 values with homogeneous steps in the logarithmic scale. For each value of $t_{\mathrm{tot}}$, 500 simulations have been done by randomly drawing the duration of the pauses. What has been noted is that the value of the dimensionless quantity $K \, \sqrt{\overline{\tau }} \, N /m_{\mathrm{ext}}$, when plotted against the dimensionless quantity *z* defined in Eq. (11), displays both an upper and a lower bound. An example is shown in Fig. 5a for $N=5$. The upper bound is found to be $\sqrt{N}$ (in accord with Eq. (6)), while the lower bound displays a *z*-dependence.

In order to investigate on the lower bound, which is ultimately connected with an upper bound on $m_{\mathrm{ext}}$, we turned to the representation of $\min \{ K \, \sqrt{\overline{\tau }} \, N /m_{\mathrm{ext}} \}$ against *z*, where the minimum value is taken from the ensemble of 500 simulations for each *z*. The results are presented in Fig. 5b for several values of *D*, *N* and $\overline{\tau }$. The unexpected outcome is that the data bundles on what looks like a “universal” profile *f*(*z*). Based on this evidence, it follows that $m_{\mathrm{ext}} \le {K \, N \, \sqrt{\overline{\tau }}}/ [1 +(\sqrt{N} - 1 ) f(z) ]$. The data were fitted with the simple three-parameter phenomenological equation $f(z) = a + (1-a)/(1+ c \, z^b)$ able to capture the qualitative features and satisfying the constraint $f(0)=1$. By fitting simultaneously the whole data set at disposal (symbols shown in Fig. 5b), we obtained $a = 0.204$, $b =1.011$, and $c = 0.594$ as best parameters. The uncertainties on the fitting parameters are not given since the adopted form of *f*(*z*) is just a trial function not based on a physical modeling. We approximated the parameters to $a = 0.2$, $b = 1$ and $c = 0.6$, as they appear in the final empirical expression Eq. (10). The profile of *f*(*z*) with such parameters is the red curve in Fig. 5b. The reason for setting $f(z) = 0$ when $z > 10$ lies in the fact that the data produced by the simulations do not allow a sound extrapolation of the profile beyond such a value of *z*; rather, by setting *f*(*z*) to zero one switches to the upper bound in Eq. (8) which is certainly valid. The utility of Eq. (9), in terms of its effectiveness in giving an estimate of the released quantity close to the true value, and possibly a precautionary overestimation, has been assessed on statistical grounds as illustrated in “Results” Section.

### Finite-thickness analysis

**Figure 6:**
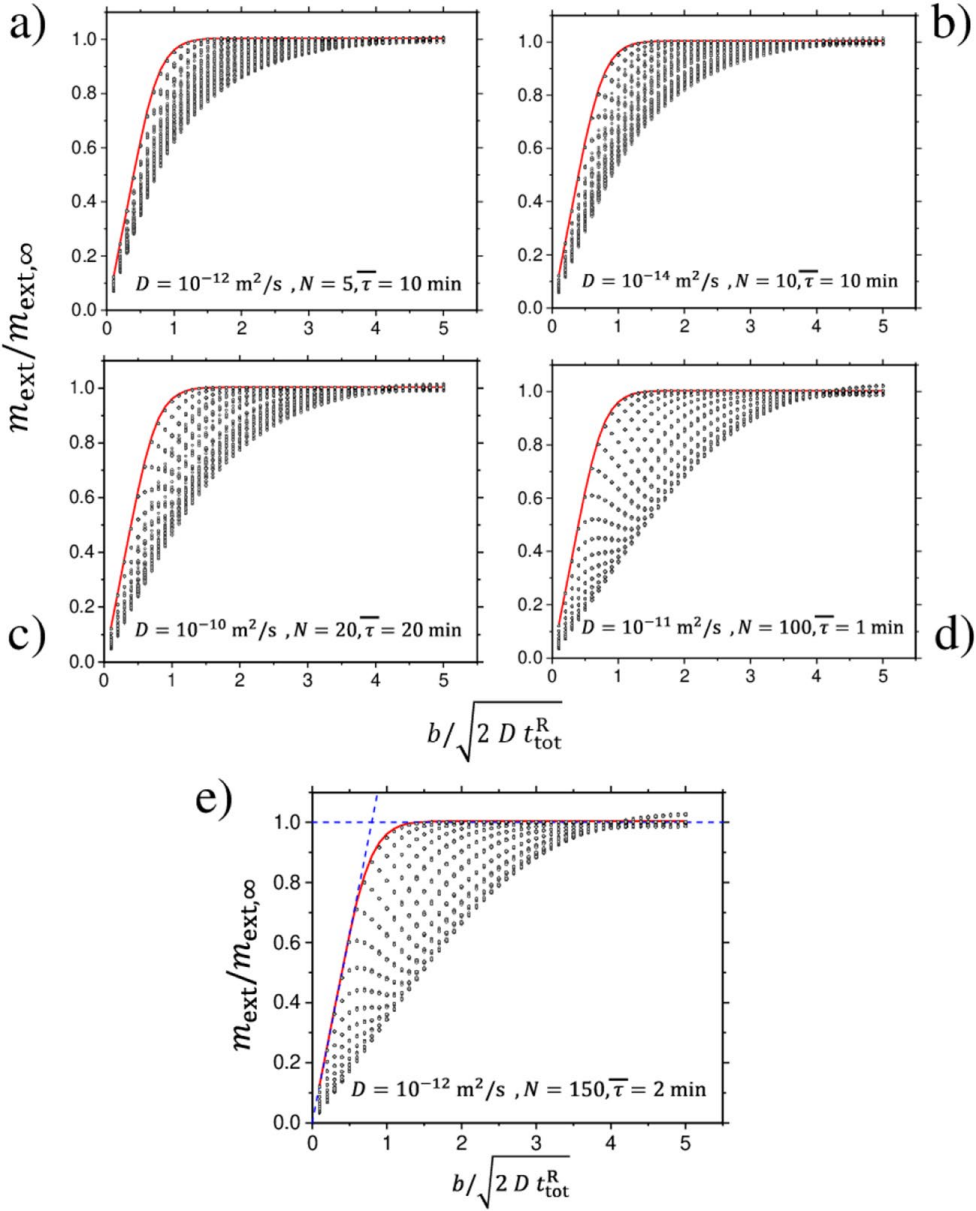
Characterization of the finite-thickness correction to $m_{\mathrm{ext}}$. Given a certain release-pause schedule, $m_{\mathrm{ext}, \infty }$ denotes the quantity released if the material had infinite thickness. Each panel refers to different values of *D*, number of release-pause phases *N*, and average release time $\overline{\tau }$. Panel (**a**): $D = 10^{-12} \; \mathrm{m^2/s}$, $N= 5$, $\overline{\tau } = 10 \; \mathrm{min}$; Panel (**b**): $D = 10^{-14} \; \mathrm{m^2/s}$, $N= 10$, $\overline{\tau } = 10 \; \mathrm{min}$; Panel (**c**): $D = 10^{-10} \; \mathrm{m^2/s}$, $N= 20$, $\overline{\tau } = 20 \; \mathrm{min}$; Panel (**d**): $D = 10^{-11} \; \mathrm{m^2/s}$, $N= 100$, $\overline{\tau } = 1 \; \mathrm{min}$; Panel (**e**): $D = 10^{-12} \; \mathrm{m^2/s}$, $N= 150$, $\overline{\tau } = 2 \; \mathrm{min}$. The continuous red lines represent the universal profile which is attained in the case of single release phase. The circles correspond to different values of the duration of the pauses. The dashed blue lines show the precautionary upper bound (see text for details).

By denoting with $m_{\mathrm{ext}, \infty }$ the amount of species released if the material had infinite thickness, and with $t^{\mathrm{R}}_{\mathrm{tot}} = N \overline{\tau }$ the total release time, its has been found an empirical correlation between the ratio $m_{\mathrm{ext}}/m_{\mathrm{ext}, \infty }$ and the ratio $b / \sqrt{2 D t^{\mathrm{R}}_{\mathrm{tot}}}$. The results are shown in Fig. 6 for several values of the parameters *D*, *N* and $\overline{\tau }$. Regardless of the specific value of the parameters, in such a representation the patterns display general features which allow to express universal bounds on $m_{\mathrm{ext}}/m_{\mathrm{ext}, \infty }$, and hence on $m_{\mathrm{ext}}$.

The continuous red line corresponds to the profile in the case $N=1$ of single release phase. For given *N*, the circles in each panel correspond to several choices of the total time $t_{\mathrm{tot}}$ (including the pause phases, see Fig. 1). In the simulations, $t_{\mathrm{tot}}$ has been varied taking 11 values between $1.1 t^{\mathrm{R}}_{\mathrm{tot}}$ and $50 t^{\mathrm{R}}_{\mathrm{tot}}$ with logarithmic progression; the thickness *b* has been varied from $\sqrt{2 D t^{\mathrm{R}}_{\mathrm{tot}}}$ to $5 \sqrt{2 D t^{\mathrm{R}}_{\mathrm{tot}}}$ taking 50 points equispaced. For each *b*, and given *N*, $t^{\mathrm{R}}_{\mathrm{tot}}$ and $t_{\mathrm{tot}}$, 10 sequences of release-pause phases have been generated at random; hence, each panel shows totally 5500 circles. Note that since *b* is divided by $\sqrt{2 D t^{\mathrm{R}}_{\mathrm{tot}}}$, in the scaled representation of Fig. 6 the specific value of the diffusion coefficient is immaterial, meaning that the same patterns would be obtained for different values of *D* keeping fixed the other parameters (this has been checked).

The outcomes indicate that the circles are spread between an upper bound, corresponding to the profile for $N =1$, and a lower bound which seems to attain a limit profile as *N* increases (further investigations are however needed to inspect such a feature). Remarkably, in all situations the distribution of the circles shrinks to the value 1 as the abscissa reaches the value of about 5. This means that, at fixed $t^{\mathrm{R}}_{\mathrm{tot}}$, the released quantity $m_{\mathrm{ext}}$ approaches $m_{\mathrm{ext}, \infty }$ when the thickness *b* is sufficiently large (in practice, when *b* is larger than $5 \sqrt{2 D t^{\mathrm{R}}_{\mathrm{tot}}}$). Note that the circles appear slightly distributed around the value 1 at such large values of the abscissa. This is only a mere numerical artifact arising from the finiteness of the discretization and amplified by error accumulation when many release-pause phases are considered. The demanding computational cost makes that the results in panel e), which have been obtained with $N_1 = 100$ and $N_2 = 200$ discretization intervals (see “Numerical solution of the diffusion equation” Section), represent the limit currently achievable. A few calculations done with thinner discretization ($N_1 = 200$ and $N_2 = 400$) have shown that the spread of the circles about the value 1 tends to be suppressed.

Concerning the upper bound, that is the profile for $N = 1$, it is found that it displays an initial linear growth with slope $\sqrt{\pi /2}$, followed by a rapid flattening to the value 1. This allows us to get a simple upper bound on $m_{\mathrm{ext}}/m_{\mathrm{ext}, \infty }$ by replacing the true profile by two straight lines with a turning point at the abscissa value of $\sqrt{2/\pi } \simeq 0.8$. The two lines are displayed as dashed blue lines in Fig. 6e. Such upper delimitation is a loose bound but has the merit to provide a simple and safe correction to Eq. (9) (which gives an approximation of $m_{\mathrm{ext}, \infty }$) when the thickness is finite. Such a correction is made explicit in “Finite-thickness correction” Section.

The characterization of the lower bound in Fig. 6 is more problematic because of the difficulty of performing calculations with very large *N*. The calculations done here, up to $N = 150$, only allow us to glimpse the convergence to a limit lower bound. Taking into account the concavity of the lower boundaries in panels from a) to e), a tentative (and loose) lower bound would be the straight line of slope 1/5. A detailed inspection of the behavior at large *N* is however required to make sound statements and get a tight lower bound.
